# Healthy Hearts – A community-based primary prevention programme to reduce coronary heart disease

**DOI:** 10.1186/1471-2261-8-18

**Published:** 2008-07-26

**Authors:** Gill Richardson, Hugo C van Woerden, Lucy Morgan, Rhiannon Edwards, Monica Harries, Elaine Hancock, Susan Sroczynsk, Mererid Bowley

**Affiliations:** 1 Caerphilly Teaching Local Health Board, Llanarth House, Unit 1 Newbridge Gateway, Bridge Street, Newbridge, Wales, UK; 2 Division of Community Health Sciences, St George's Medical School, University of London, 6/F, Hunter Wing, Cranmer Terrace, London, UK

## Abstract

**Background:**

The ten year probability of cardiovascular events can be calculated, but many people are unaware of their risk and unclear how to reduce it. The aim of this study was to assess whether a community based intervention, for men and women aged between 45 and 64 years without pre-existing coronary heart disease, would reduce their Framingham scores when reassessed one year later.

**Methods:**

Individuals in the relevant age group from a defined geographical area were sent an invitation to attend for an assessment of their cardiovascular risk. Individuals with pre-existing cardiovascular disease or terminal illness were excluded. The invitation was in the form of a "Many Happy Returns" card with a number of self-screening questions including the question, "If you put the enclosed string around your waist, is it too short?" The card contained a red 80 cm piece of string in the case of women, or a green 90 cm piece of string in the case of men. At the assessment appointment, Framingham scores were calculated and a printout was given to each individual. Advice was provided for relevant risk factors identified using agreed guidelines. If appropriate, onward referral was also made to a GP, dietician, an exercise referral scheme, or to smoking cessation services, using a set of guidelines. Individuals were sent a second invitation one year later to return for re-assessment.

**Results and discussion:**

2031 individuals were asked to self-assess their eligibility to participate, 596 individuals attended for assessment and 313 of these attended for follow-up one year later. The mean reduction in the Framingham risk score, was significantly lower at one year (0.876, 95% CI 0.211 to 1.541, p = 0.01). The mean 10-year risk of CHD at baseline was 13.14% (SD 9.18) and had fallen at follow-up to 12.34% (SD 8.71), a mean reduction of 6.7% of the initial 10-year Framingham risk. If sustained, the estimated NNT to prevent each year of CHD would be 1141 (95% CI 4739 to 649) individual appointments.

**Conclusion:**

This community intervention for primary prevention of CHD reduces Framingham risk scores at one year in those who engage with the programme.

## Background

A range of risk factors for coronary heart disease (CHD) have been identified and the probability of adverse cardiovascular events over the subsequent 10 years can be predicted using tools such as the Framingham Risk Score [1]. The risk of cardiovascular disease can be modified by a number of behavioural changes and by treatment of undiagnosed hypertension and lipid disorders. However, many individuals are unaware of their level of risk and do not have access to information that might influence their health behaviour.

A case can be made for identifying 'at risk' individuals, particularly over 45 years of age and for providing them with advice on reducing their risk. There is good evidence that secondary prevention interventions are effective [2,3] but there is less evidence for interventions aimed at primary prevention of CHD. Most General Practitioners (GPs) undertake opportunistic screening and have compiled CHD registers. However, many individuals are not identified by this opportunistic screening, either because they have not attended their GP, have not been opportunistically assessed when visiting their GP, or perhaps have not been identified as 'at risk' of CHD because they avoid contact with health service providers. Universal screening of the whole population is not merited as the risk of CHD rises with age and the great majority of individuals younger than 45 years have a low risk of CHD in the subsequent 10 years. This study consequently encouraged a degree of self selection using a range of self assessment criteria, although patients not meeting these criteria were not rigidly excluded.

Consequently, the aim of this study was to assess whether a community based intervention, for men and women aged between 45 and 64 years without pre-existing coronary heart disease, would reduce their Framingham risk scores when reassessed one year later. A secondary aim of this study was to assess which risk factors had changed most over the course of the year and so contributed most to any reduction in Framingham risk scores.

## Methods

Men and women aged between 45 and 64 years and registered with three GP practices in the Rhymney Valley, Wales were identified. The practices had 4,672 patients registered with them in the relevant age group. This area was chosen because it has a high level of social deprivation and a high Standardised Mortality Rate for CHD. Individuals who had pre-existing cardiovascular disease and were on the practices' CHD registers were identified and excluded, as were patients with known terminal disease. Each week between September 2004 and March 2005, approximately 200 individuals in the relevant age group were sent an invitation to self-screen for eligibility to attend for an assessment of their risk of heart disease until all eligible patients in the GP practices had been invited. The invitation was in the form of a "Many Happy Returns" card containing a number or questions and enclosing a red 80 cm piece of string in the case of women, and enclosing a green 94 cm piece of string in the case of men. The invitation card, which was intended to stimulate reflection on CHD risk factors, included the following: "Have you ever been told you have high blood pressure? Have you ever been told you have high cholesterol? Do you smoke now or have you smoked in the past 15 years? Has your mother, father, brother or sister ever had heart problems, before they were 60 years old? If you put the enclosed string around your waist, is it too short? You could benefit from a free health check to help prevent heart problems if you ticked any of the 'yes' boxes". The card invited eligible individuals to phone and make an appointment for the assessment. A single reminder was sent out two weeks after the first invitation. When individuals telephoned the free telephone number provided, a range of dates were offered over the subsequent four weeks for the assessment to be undertaken.

The assessment clinics were held in community venues not normally associated with health care. At the clinic, individuals were provided with an information sheet, filled in a number of questionnaires, and signed a consent form allowing the data gathered to be used for research purposes. Framingham scores were calculated using a computer programme called CV-R Profile [4]. This produced a printout, which was given to each individual seen. Advice was provided for relevant risk factors identified using agreed guidelines. Where relevant, onward referral was also made to the individual's GP, a dietician, an exercise referral scheme, or to local smoking cessation services, using a set of guidelines based on published sources [5-7]. Some individuals were referred to more than one source of subsequent advice or assessment. Satisfaction questionnaires were completed by individuals at the end of their assessment. Individuals that attended were sent a second invitation one year later to return to an identical clinic for re-assessment of their Framingham risk score. Ethical approval for the study was obtained from the local NHS research ethics committee.

The study was developed by a group of public health staff in Caerphilly Local Health Board (LHB) with advice from academic colleagues as a bid for funding to address "inequalities" in Caerphilly LHB. It was piloted on staff in the LHB and in a neighbouring cottage hospital. The experience gained was used to make changes that were incorporated into the final design.

Data was analysed using SPSS 14. The paired t-test for 'the difference between means for paired samples' was used where data was continuous and parametric, and the Wilcoxon Signed-Rank Test, Mann-Whitney Test, or Chi-square test was used elsewhere.

## Results

**Figure 1:**
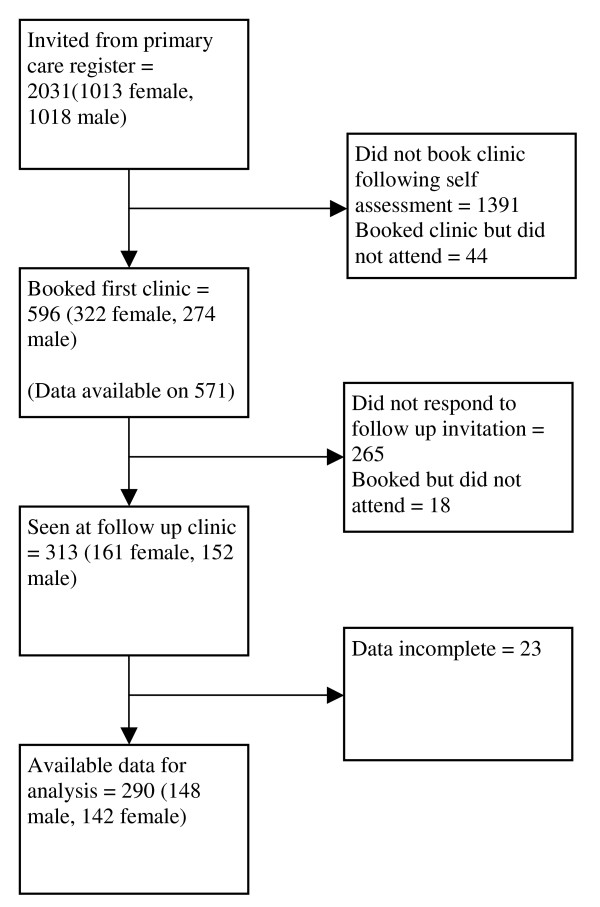
Flow diagram of study participants.

Of the 2,031 individuals invited to assess their eligibility, 596 individuals attended for assessment and 313 of these attended for follow-up one year later (Figure 1). Consent was obtained and data was available for analysis on 290 individuals (148 men and 142 women) who did re-attend for follow-up and on 281 individuals who did not re-attend for follow-up (Table 1). Participants were almost all of Caucasian origin, reflecting the ethnicity of the local population. The 'Did Not Attend' (DNA) rate for those who had made appointments was 7.4% for the initial visit and 5.8% for the follow-up visit.

**Table 1:** Study participants by age and gender

**Age band**	**Female**	**Male**	**Total**
45–49 yrs	21	22	43
50–54 yrs	36	34	70
55–59 yrs	47	37	84
60–64 yrs	44	49	93
Total	148	142	290

The main outcome of the study, the mean reduction in the Framingham risk score, was slightly lower at one year (0.876%, 95% CI 0.21% to 1.54%). The mean 10-year risk of CHD at baseline was 13.14% (SD 9.18) and had fallen at follow-up to 12.34% (SD 8.71), a mean reduction of 6.7% of the initial 10-year Framingham risk.

**Figure 2:**
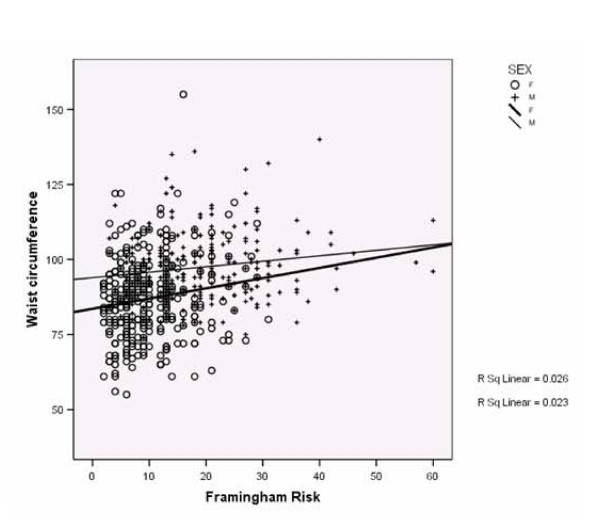
Scatter plot of Framingham score (10-year risk of CHD) against waist circumference (cm).

Table 2 indicates that weight, BMI and waist circumferences worsened at follow-up. However, other characteristics: pulse, systolic BP, total cholesterol, HDL, and glucose profiles significantly improved, even accounting for the multiple statistical comparisons undertaken. Figure 2 indicates that waist circumferences correlated with Framingham risk scores in both men and women but with a wide spread of results.

**Table 2:** Changes in risk factors from baseline to follow-up at one year for study participants

**Improved**	**Metric**	**Mean**	**Std. Deviation**	**Sig. (2-tailed)**
	Baseline Weight (kg)	78.95	16.735	
1. No	Follow-up Weight (kg)	79.367	16.8307	0.023
	Baseline BMI	28.13	4.843	
2. No	Follow-up BMI	28.38	4.872	0.001
	Baseline Waist circumference (cm)	89.56	17.027	
3. No	Follow-up Waist circumference (cm)	90.94	13.656	0.059
	Baseline Systolic BP (mmHg)	141.53	18.681	
4. Yes	Follow-up Systolic BP (mmHg)	138.65	17.014	<0.001
	Baseline Diastolic BP (mmHg)	83.78	9.606	
5. Yes	Follow-up Diastolic BP (mmHg)	82.65	10.449	0.022
	Baseline Pulse Pressure	57.75	13.873	
6. Yes	Follow-up Pulse Pressure	55.93	12.745	0.003
	Baseline Total Cholesterol	5.5113	1.04420	
7. Yes	Follow-up Total Cholesterol	5.3530	.99085	0.002
	Baseline HDL (mmol/l)	1.2777	.37721	
8. Yes	Follow-up HDL (mmol/l)	1.3787	.40722	<0.001
	Baseline Glucose (mmol/l)	5.4884	.90338	
9. Yes	Follow-up Glucose (mmol/l)	5.305	1.1556	0.008

Assessment of relevant health behaviours indicated that, at follow-up, smoking had decreased, alcohol intake had decreased, and regular exercise had increased, although the extent of the changes observed was not statistically significant. The greatest behavioural change was in reported intake of fruit and vegetables, where the increase was statistically significantly (Wilcoxon signed rank test, p > 0.001).

### Groups with the greatest improvement

Three factors were significantly associated with being in the one third of individuals who had the largest reductions in Framingham risk score at one year: higher initial Framingham risk score, referral onwards to another service (for further advice, assessment, treatment or health promoting activity), and older age.

Individuals who had been referred onwards to another service for further advice had a large fall in their mean Framingham risk score (1.869%), whereas those who were not referred onwards had very little fall in their Framingham risk score (0.155%). The mean difference in the change of risk scores between those who were referred onwards and those who were not was 1.71% (95% CI 0.38% to 3.05%, p = 0.012). As would be expected, those referred onwards had higher initial Framingham risk scores.

Details of the nature of the 169 onward referrals, made on 122 individuals, are shown in Table 3. One individual had four onward referrals, four individuals had three onward referrals, 36 individuals had two onward referrals and 81 individuals had one onward referral. Onward referral had an effect on reducing smoking but not on exercise, alcohol intake or fruit and vegetable intake (Table 4).

**Table 3:** Type and number of onward referrals

**Type of referral**	**Referrals**
Cardiac Rehabilitation	1
GP/nurse re blood pressure	52
GP/nurse re cholesterol	83
GP/nurse re other concerns	17
GP/nurse re exercise	0
Dietician re weight	7
Smoking Cessation	4
Exercise Referral via a "Health Living" Centre	3
Dietician Referral re cholesterol	2

Total	169

**Table 4:** The effect of onward referral on changes in behaviours to reduce CHS risk at one year follow-up

	**Not Referred onwards**		**Referred onwards to any service provider**		**Chi-square p value**
			
	**Risk factor reduced**	**Risk factor increased**	**Risk factor reduced**	**Risk factor increased**	
Smoking	11	15	19	8	0.04
Exercise	62	45	37	36	0.33
Alcohol	37	36	22	21	0.96
Fruit and veg intake	63	30	40	25	0.42

*Cases where there has been no change in behaviour have been excluded

Table 5 indicates that those who were in older age groups were significantly more likely to be in the one third of individuals with the greatest improvement in Framingham score (Chi square, p = 0.043).

**Table 5:** Change in Framingham score by age group when reviewed after one year

**Change in Framingham scores (%)**	**45–49 yrs**	**50–54 yrs**	**55–59 yrs**	**60–64 yrs**	**Total**
Third with greatest improvement %	15	20	40	46	121
	34.9%	28.6%	47.6%	49.5%	41.7%
Middle third %	10	28	22	21	81
	23.3%	40.0%	26.2%	22.6%	27.9%
Third with least improvement %	18	22	22	26	88
	41.9%	31.4%	26.2%	28.0%	30.3%
Total (individuals in age band) %	43	70	84	93	290
	100.0%	100.0%	100.0%	100.0%	100.0%

Chi square, p = 0.043. Thirds are not identical in size as the data is ordinal and can therefore only be approximately split into three groups.

A significant difference was noted in this study between men and women in the reporting of a family history of heart disease, although most men and women were indigenous to the area and therefore drawn from the same extended family networks. A family history of CHD was reported by 33.5% of women, but only 15.0% of men (Chi square p < 0.001).

Evaluation forms, completed by 452 individuals after attending their initial assessment appointment, indicated that 98% found the appointment 'very useful', 1.3% 'useful', 0.2% 'not very useful' and 0.4% did not complete the evaluation question. The venue used was considered 'suitable' by 96% as against 3.8% who considered it 'unsuitable'.

### Comparison with those who did not re-attend

A number of differences were identified in the baseline characteristics between those who returned for follow-up at one year and those who did not. The mean Framingham risk score for those who did not return for follow-up (14.7%) was slightly higher than the mean risk score for those who did return (13.2%). In addition, those who returned for follow-up were, on average, 1.23 years older than those who did not return for follow-up. Men were also slightly more likely to return for follow-up than women (52.8% cf. 49.0%).

Using the mean reduction in Framingham risk score of 0.876% over 10 years, suggests a number needed to treat (NNT) of 114.1 to prevent one person developing heart disease over 10 years, or 1141 (95% CI 4,739 to 649) individual appointments to prevent each year of CHD.

Making the conservative assumption that out of the 2,031 individuals invited, improvement only occurred in the 313 who returned for re-assessment at one year, an invitation would need to be sent by the programme to 7,407 individuals (95% CI 4,202 to 30,769) to prevent one year of CHD in one notional individual. At an estimated cost of at least £20 per individual seen, this equates to at least £150,000 to prevent one year of CHD in one notional individual.

## Discussion

This study's main finding is that a community based programme inviting men and women aged between 45 and 64 years, who do not have pre-existing coronary heart disease, assessing their 10-year risk and providing appropriate advice or onward referral related to risk factors identified, slightly reduced the subsequent risk of CHD, as measured by Framingham scores one year later. The magnitude of the mean reduction in the estimated 10-year risk of developing cardiovascular disease was 0.876% against a mean initial 10-year estimated risk of CHD of 13.14% in this population. Based on our reading of the literature, our aim was to reduce the average Framingham risk score by 7%. The reduction we achieve was slightly smaller than this (6%). However, the clinical significance of a less than one percent absolute change in Framingham Risk Score is not clear.

There was some evidence that the change which was observed may have been mediated through onward referral for a number of further interventions including: further assessment, reinforcing advice, or relevant prescription of medication. Those at greatest risk and those who were older at the time of assessment seem to have benefited most. There was some evidence that the intervention had improved self-reported behaviours around diet, exercise, smoking and alcohol consumption.

The intervention positively impacted on diet, exercise, alcohol intake and smoking behaviour but did not appear to have a beneficial impact on obesity and further work is needed to address this risk factor [8].

Blood pressure and lipid levels were also improved, although the size of the observed fall in blood pressure was similar to that which has been observed as a result of regression towards the mean, or a placebo effect, displayed in the control groups of some clinical trials. The adverse BMI and weight changes are not directly reflected in the Framingham risk score and have, therefore, not been reflected in the fall in mean risk scores. It is unclear whether the observed increases in weight would have adversely affected the estimated risk of CHD in such individuals.

The study is not representative of areas with lower rates of CHD nor would it be generalisable to areas with low rates of CHD. The GP practices from which individuals were invited were in an area of high rates of CHD. Standardised Mortality Ratios (SMR) for the area, extracted from the Welsh Public Health Common Data Set for the five years 1995–99, for the wards from which these patients were drawn were: New Tredegar (134.2), Darren Valley (126.4), Moriah (119.2), Pontlottyn (126.0). The GP practices used were the main providers of primary care to the above wards and were, therefore, representative of the patients living in this area. The study does demonstrate the potential feasibility of reducing "inequalities" in some CHD risk factors in communities with very high rates of CHD.

This study was undertaken in a service setting, rather than a purely academic context, and should therefore be relatively easy to replicate. The most significant weakness in the study has been the lack of a control group although we hope to measure differences in the mortality rates between participating practices in the target area and non participating practices in an adjacent area. However, as this information is not available at an individual patient level, it will be open to the ecological fallacy. We do not have comparable data on non-participants from the relevant GP practices, as it was considered unethical to use the GP surgery databases to obtain information on patients without their consent.

A significant proportion of those invited to a follow-up appointment one year later did not choose to re-attend, and this group appears to have had a higher average risk of CHD, a finding that has been replicated elsewhere [9]. Younger individuals in the cohort were also less likely to re-attend for assessment. However, the interventions appears to have been well received as evaluation immediately after the initial appointment indicated that over 99% of all attendees had found the content of the assessment 'useful'.

Some concerns have been expressed that the Framingham algorithm was used in this study [1] may over estimate risk [10] or underestimate the risk [11]. However, it has been widely used in CHD research and clinical practice to communicate risk to individuals and is easily understood and calculated.

The confidence intervals used to assess the changes in the mean differences in Framingham scores, in the results provided, are also open to the criticism that they do not take into account the underlying variance arising from the different risk factors incorporated into each individual's specific risk estimate. The true standard deviations and confidence intervals for the mean Framingham risk scores provided for different groups may, therefore, be larger than those that have been presented.

A number of studies and systematic reviews [12-14] have assessed multifaceted interventions to reduce the risk of CHD and have demonstrated reductions in risk factors, although not necessarily in mortality rates. The effectiveness of these interventions appears to be greatest in those at greatest risk of CHD. However, it is difficult for individuals to assess their risk of CHD without information on their lipid profile, which requires blood sampling. This suggests that there is a need for tools that individuals could use themselves as an initial screening measure before further clinical assessment. This study used a set of self-administered questions inside a card as an initial 'screening tool', which meant that those attending for the calculation of a Framingham risk score were at increased risk of CHD. However, there is the potential to refine the set of self-completed questions in the initial invitation and further differentiate those at very low risk and who are least likely to benefit from further assessment, from those at higher risk who would benefit from further assessment.

A piece of string was included with instructions to use it as a 'screening tool' to assess their own abdominal circumference based on previous research which indicates that abdominal circumference predicts CHD risk [15]. Using "waist circumference greater than the piece of string provided" as a 'screening test', to identify those individuals with a Framingham risk of 10% or greater, would have had a sensitivity of 75.4% in women and 76.1% in men within the cohort who attended our clinics. Using this criterion on its own, to invite individuals for further assessment, would have missed a significant proportion of those with a Framingham risk of greater than 10%.

There are a number of areas where further work could be undertaken to improve and build on this intervention. Individuals varied widely in the effect of the intervention. There is a need to explore the varying experiences of individuals involved in the study, particularly those whose scores had changed most radically to assess those aspects of the intervention that had most influenced them. It may also be possible to further enhance participation rates, effectiveness and impact of advice given, for example by using motivational interviewing. The screening questions in the invitation card could also be modified to increase the effectiveness of the self-assessment as a screening tool.

## Conclusion

This community intervention for primary prevention of CHD reduces Framingham risk scores at one year in those who engage with the programme. It is unclear to what extend this effect is sustained in the longer term although follow up is ongoing. There may also be scope for further refining the intervention to target those individuals that are at highest risk of CHD but have not previously been identified by other means.

## Competing interests

The authors declare that they have no competing interests.

## Authors' contributions

GR initiated the study, led design of the project, and secured and managed the resources; HCvW undertook the statistical analysis used in this paper and wrote the draft; LM established and maintained the database; SS, RE, and HCvW led on obtaining ethical approval; all the other contributors undertook practical aspects of the project and/or reviewed the draft paper.

## Pre-publication history

The pre-publication history for this paper can be accessed here:


